# A new assay based on terminal restriction fragment length polymorphism of homocitrate synthase gene fragments for *Candida* species identification

**DOI:** 10.1007/s13353-017-0394-5

**Published:** 2017-03-27

**Authors:** Kasjan Szemiako, Anna Śledzińska, Beata Krawczyk

**Affiliations:** 1 Department of Molecular Biotechnology and Microbiology, Faculty of Chemistry, Gdańsk University of Technology, Narutowicza 11/12, 80-233 Gdańsk, Poland; 2 Department of Therapy Monitoring and Pharmacogenetics, Medical University of Gdańsk, Gdańsk, Poland

**Keywords:** *Candida*, t-RFLP, PCR, Homocitrate synthase gene, Molecular diagnostic

## Abstract

*Candida* sp. have been responsible for an increasing number of infections, especially in patients with immunodeficiency. Species-specific differentiation of *Candida* sp. is difficult in routine diagnosis. This identification can have a highly significant association in therapy and prophylaxis. This work has shown a new application of the terminal restriction fragment length polymorphism (t-RFLP) method in the molecular identification of six species of *Candida*, which are the most common causes of fungal infections. Specific for fungi homocitrate synthase gene was chosen as a molecular target for amplification. The use of three restriction enzymes, *Dra*I, *Rsa*I, and *Bgl*II, for amplicon digestion can generate species-specific fluorescence labeled DNA fragment profiles, which can be used to determine the diagnostic algorithm. The designed method can be a cost-efficient high-throughput molecular technique for the identification of six clinically important *Candida* species.

## Introduction

Fungal infections of the *Candida* sp. etiology are a serious clinical problem. The mortality of candidemia episodes exceeds 50%. Furthermore, there are many factors which make this infection more and more dangerous, such as development of drug resistance, immunosuppressive therapies, diabetes, and increasing number of cancer incidence (López-Martínez 2010). The treatment of infection varies depending on its etiological agents (Miceli et al. 2011). This is the main reason why rapid and accurate diagnosis is needed. Natural antibiotics resistance differs among *Candida* species, which makes it crucial to know which species are going to be dealt with (e.g., fluconazole resistance of *C. glabrata* and *C. krusei*, which is the first-line antifungal treatment) (Tortorano et al. 2004). Correct therapy based on fast and reliable diagnosis is essential to treat patients successfully and to decrease mortality.

Opportunistic fungal infections in immunocompromised hosts are caused mainly by *Candida* species, and the majority of such infections are due to *C. albicans* (Silveira-Gomes et al. 2011), which shares many phenotypic features with *C. dubliniensis*, and may, therefore, be misidentified in clinical microbiology laboratories. Species-specific differentiation of the two closely related yeasts, *C. albicans* and *C. dubliniensis*, is difficult in routine diagnosis. Reliable, routinely applicable methods for species-specific differentiation of *C. albicans* and *C. dubliniensis* appear to be of particular importance to better understand the epidemiology and virulence of *C. dubliniensis* (Hof et al. 2012). Candidemias caused by *C. dubliniensis* have been increasingly reported in recent years (Ahmad et al. 2012).

Methods for the identification of *Candida* sp. can be divided into conventional and molecular techniques. Conventional methods are based on the germ tube test, chlamydospore formation, and the fermentation or assimilation of sugars (Alam et al. 2014). There are also several chromogenic media for the isolation and identification of *Candida* species (Letscher-Bru et al. 2002). Colonies growing on these media have different morphology and color as a result from the cleavage of chromogenic substrates by species-specific enzymes (Bauters and Nelis 2002). However, the colors of colonies of one species may differ depending on the strain and may lead to misidentification (Ozcan et al. 2010). Methods for *Candida* identification can also be divided into non-DNA- and DNA-based techniques. In the group of non-DNA-based methods, there are such identification tests as serological [latex agglutination, enzyme-linked immunosorbent assay (ELISA), immunoblotting, dot immunoassay, liposomal immunoassay, and radioimmunoassay (RIA) (Ponton et al. 2002)] and spectroscopic methods (Manzoor et al. 2016). A very large and still fast developing group of methods are DNA-based techniques. They are mostly based on different types of polymerase chain reaction (PCR): microsatellite typing (Zane et al. 2002), multilocus sequence typing (MLST) (Odds 2010), randomly amplified polymorphic DNA (RAPD) (Melo et al. 1998), simplex PCR (Trtkova and Raclavsky 2006), multiplex PCR (Lau et al. 2008), nested PCR (Kanbe et al. 2002), real-time PCR (Fricke et al. 2010; Olchawa et al. 2013), and restriction fragment length polymorphism (RFLP) (de Llanos Frutos et al. 2004). The latter method as the terminal restriction fragment length polymorphism (t-RFLP) method is used for the genotyping and study of microbial diversity and microorganisms community structure in different environmental samples (Osborn et al. 2000; Schütte et al. 2008; Zhang et al. 2008; Waldron et al. 2009; Caretta and Brito 2011). This method is a modified classical PCR with a fluorescently labeled primer(s) and is linked to the digestion of amplicons with restriction enzyme(s). Only terminal fragments of PCR product containing fluorescent dye are visualized.

The aim of this study was to elaborate a method to differentiate six *Candida* species using the PCR t-RFLP technique based on a novel molecular target, the homocitrate synthase gene. The homocitrate synthase gene encodes an enzyme involved in the first reaction in the lysine biosynthesis pathway. The protein is characteristic for fungi and several Archaea and two isoforms are present for some *Candida* species. This gene is considered as a good molecular target for chemotherapy of disseminated fungal infections. It was the prerequisite for the selection of this gene as a novel molecular target for *Candida* species identification (Kur et al. 2010).

## Materials and methods

### *Candida* strains

In this study, the strains collection encompassed 75 clinical and six reference strains of *Candida* sp. The reference strains were as follows: *C. albicans* ATCC 64544, *C. krusei* ATCC 6258, *C. glabrata* ATCC 2001, *C. parapsilosis* ATCC 22019, *C. tropicalis* ATCC 750, and *C. dubliniensis* ATCC MYA-646. Clinical strains were collected from patients of Public Hospital No. 1 in Gdańsk, Poland and were identified according to the routine laboratory procedure. Strains were isolated from blood cultures (3), respiratory tract (28), urine (20), and genitourinary tract (from swabs) (24). In case of patients with suspected candidemia, blood was cultured using BacT/ALERT 3D (bioMérieux) for about 5 days. The species identification procedure involved subculture plates incubation on Sabouraud dextrose agar (SDA, bioMérieux), identification of isolated strains on chromogenic media for *Candida* (CHROMagar® *Candida*), and the biochemical panel VITEK® 2.

### Culture and DNA isolation

Yeasts were cultivated in Sabouraud media with chloramphenicol (Emapol, Poland) for 24 h at 37 °C. The DNA isolation from the single colony was performed using the ExtractMe Purification Kit (Blirt DNA-Gdańsk, Poland), according to the manufacturer’s recommended procedure. The DNA concentration was measured using a NanoDrop ND-100 spectrophotometer (Thermo Fisher Scientific, Wilmington, USA) and ranged from 10 to 60 ng per microliter.

### PCR

Based on the homocitrate synthase gene sequences available in the NCBI GenBank (*C. albicans* XM_708526, XM_708489, XM_707839, *C. dubliniensis* XM_002418342, XM_002417072, *C. glabrata* XM_447985, XM_448111, *C. tropicalis* XM_002547242, XM_002550342, and *C. parapsilosis* HE605206-3, HE605203), oligonucleotide primers for PCR reaction were designed and their specificity was tested using, in both cases, the BLAST sequence tool (NCBI Blast). There were no sequences for *C. krusei* available and these sequences were obtained during this study (*C. krusei* KT_362370, KT_362371). PCR reaction was carried out using the standard protocol at a volume of 50 μL 0.2 mM of each dNTP (Blirt DNA-Gdańsk, Poland), 2 mM of MgCl_2_ (Blirt DNA-Gdańsk, Poland), 4 μM of each primer, the forward primer was labeled with fluorescein [5(6)FAM] at the 5′ end [LYSfor: 5′-5(6)FAM-AGAGAAGGTGAACAATTTGC-3′ and LYSrev: 5′-CCAACAGTATCAGCAATACCAACTCT-3′, Metabion, Germany], 1 U of *Pfu* Polymerase Hypernova (Blirt DNA-Gdańsk, Poland), and 1 μL of fungal DNA. The reaction profile was as follows: 94 °C for 120 s initial denaturation, 94 °C for 30 s, 59 °C for 30 s, and 72 °C for 30 s for 30 cycles, and then a final extension at 72 °C for 300 s.

### Digestion of amplicons

Restriction analysis of each amplicon was carried out with three endonucleases (Thermo Fisher Scientific, USA), *Rsa*I, *Dra*I, and *Bgl*II, as separate reactions. Restriction enzymes were chosen using the NEBcutter tool (New England Biolabs; http://tools.neb.com/NEBcutter/index.php3). They were optimized to obtain the most differentiation potential for distinguishing six *Candida* species using minimal types of enzymes. Digestion of amplicons was carried out in 20 μL [15 μL of PCR sample, 2 μL of 10× reaction buffer (Thermo Fisher Scientific, USA), 0.5 μL (2 U/μL) of endonuclease, and 2.5 μL of water] for 40 min at 37 °C. Digested products were separated by electrophoresis in homemade 12% polyacrylamide gel. There was no ethidium bromide in gel; therefore, only fluorescence from fluorescein-labeled primers was observed in UV light.

## Results and discussion

The technique that forms the basis of the described method has become widely used in microbial community studies. Molecular methods are especially useful for culture-difficult and uncultivated microorganisms (Siqueira 2017). Microbial diversity studies involving the t-RFLP method investigate not only health and diseases influences (Jung et al. 2016), but also industry issues (Hedrich 2016). The most commonly used molecular targets are sequences within the ribosomal operon (Hayashi et al. 2014). In contrary to the references in this work, a novel application of the t-RFLP method is shown. The selection of specific molecular targets other than ribosomal DNA and designing the method for species identification shows the new potential of the t-RFLP method.

In this study, we apply the t-RFLP method for the identification of the six medically important *Candida* species using the universal primers for homocitrate synthase gene fragments. The length of PCR products for all *Candida* tested was 470 bp. It was established in silico using the NCBI Primer Blast tool. A set of three restriction endonucleases (*Rsa*I, *Dra*I, and *Bgl*II) for differentiation of the investigated *Candida* species was chosen. Optimization of the number and kind of enzymes was carried out based on the NEBCutter tool. Two genes, LYS21 and LYS22, encoding isoforms of homocitrate synthase, are present and, for each copy, we can get a different pattern of digestion or not. The results of theoretical restriction patterns are presented in Table 1.

**Table 1:** Theoretical restriction fragments for *Candida* species. The numbers in **bold** represent the first restriction fragments from the 5′ end of the leading strand of polymerase chain reaction (PCR) products. These fragments contain fluorescently 5′ labeled primer and are visualized in electrophoresis. The other numbers represent the rest of the restriction fragments obtained after the digestion of PCR products (they are not visualized after electrophoresis). In some cases, there are two sets of restriction patterns for one species. This appears when the DNA sequences of enzyme isoschizomers gene polymorphism results in different recognition site positions

Species	Endonuclease		
	*Bgl*II (bp)	*Rsa*I (bp)	*Dra*I (bp)
*C. albicans*	470*	420*/50	161*/309
*C. dubliniensis*	470*	420*/50	161*/309
	385*/85		
*C. tropicalis*	470*	255*/164/51	**161**/309
	385*/85		
*C. glabrata*	470*	255*/193/22	470*
*C. parapsilosis*	470*	420*/50	470*
	171*/299		
*C. krusei*	470*	255*/164/51	161*/309

*Fragment labeled with fluorescence as a visible band on electrophoretic gel

**Fig. 1:**
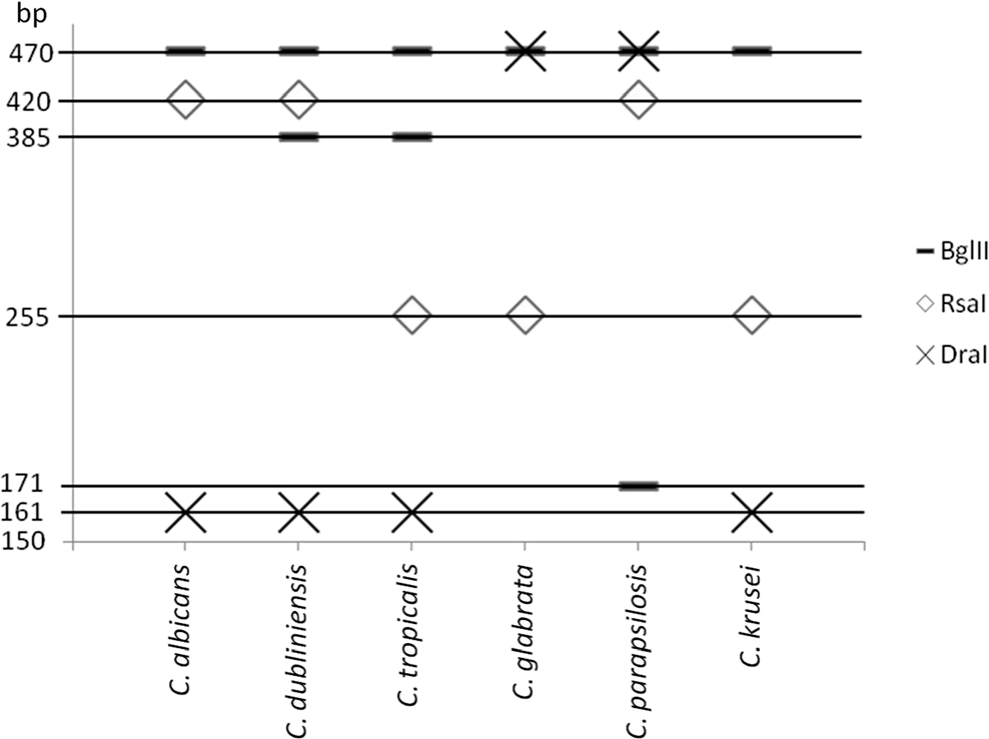
Theoretical restriction patterns after electrophoresis for each species. *Rsa*I, *Bgl*II, and *Dra*I are the restriction enzymes. Each icon described in the legend represents one restriction enzyme, for which restriction products that should appear in gel after electrophoresis are visualized. The length of restriction products with fluorescently labeled primers is represented by the *y*-axis

**Fig. 2:**
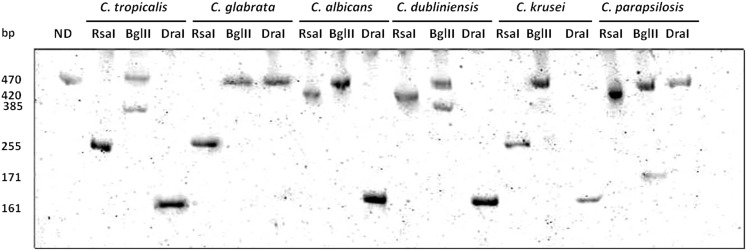
Electropherogram of digestion products obtained for the six reference *Candida* strains. Electrophoresis was carried out in 12% polyacrylamide gel for 3 h (8 V/cm). There was no dye in the gel. Visualization of DNA fragments was carried out in UV light. *ND* polymerase chain reaction (PCR) product without digestion; *Rsa*I, *Bgl*II, and *Dra*I are the patterns of digestion for restriction enzymes

We have shown discriminatory power for these restriction enzymes to distinguish the particular *Candida* species (Figs. 1 and 2). They have the potential for differentiation of six clinically important *Candida* species. Additionally, the digestion step can be modified for specific needs. Depending on how many and which species must be identified, the optimal steps of digestion by different enzymes and their order may be chosen. For example, the identification of *C. parapsilosis* sensu lato may be realized with only one *Bgl*II enzyme. In the case of *C. albicans* and *C. dubliniensis*, two *Rsa*I and *Dra*I enzymes can be used for identification. The need to use all three restriction enzymes is only applicable when all six of the investigated *Candida* species may be expected in a sample. The presence of a 470-bp band indicates that there is a lack of amplicon digestion. An appearance of another fragment in the presence of a 470-bp band for the particular enzyme indicates that the second copy of this gene has a restriction site.

**Fig. 3:**
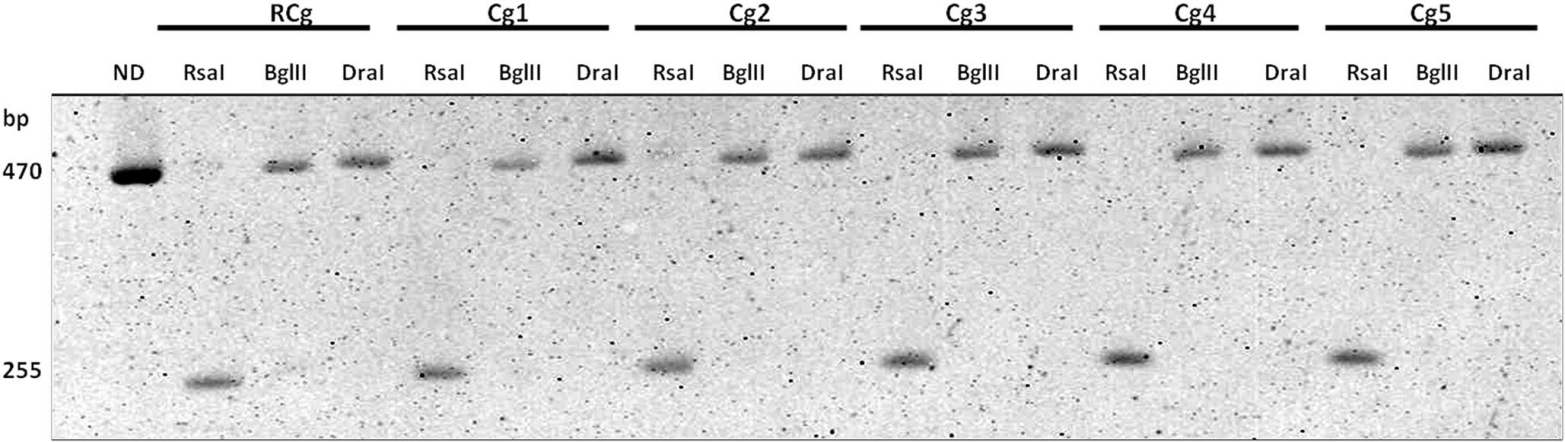
Representative electropherogram obtained for clinical strains of *Candida glabrata* using the terminal restriction fragment length polymorphism (t-RFLP) PCR method. Electrophoresis was carried out in 12% polyacrylamide gel for 3 h (8 V/cm). There was no dye in the gel. Visualization of DNA fragments was carried out in UV light. *ND* PCR product without digestion of restriction enzyme(s); *RCg* *C. glabrata* ATCC 2001 reference strain; *Cg1*, *Cg2*, *Cg3*, *Cg4*, *Cg5* *C. glabrata* clinical strains; *Rsa*I, *Bgl*II, and *Dra*I are the restriction enzymes

The established diagnostic algorithm for reference strains was confirmed with clinical strains (Fig. 3). All clinical strains were identified correctly compared to results obtained using CHROMagar. Clinical strains identified by the t-RFLP method and clinical sources are presented in Table 2. However, very often, phenotyping methods like CHROMagar create problems with determining the color and giving an accurate identification, so choosing the appropriate therapy is impossible. All digestion reactions must be carried out in separate tubes because simultaneous digestion with three endonucleases in one reaction results in obtaining only the shortest product, which does not identify the species. However, different restriction patterns allows to mix reaction mixtures for all three nucleases after digestion and separate such mixtures using electrophoresis (*C. albicans* three bands 470, 420, and 161; *C. dubliniensis* four bands 470, 420, 385, and 161; *C. tropicalis* three bands 470, 385, and 161; *C. glabrata* two bands 470 and 255; *C. parapsilosis* three bands 470, 420, and 171; and *C. krusei* three bands 470, 255, and 161) (Fig. 1). Digested products may also be separated by electrophoresis in 3–4% high-resolution agarose gel (agarose HiRes grade, Bioline) for 90 min (7 V/cm), but the detection sensitivity decreases and, therefore, more products of the digestion reaction should be applied.

**Table 2:** Clinical strains identified using the terminal restriction fragment length polymorphism (t-RFLP) method

Species	Clinical sample			
	Blood cultures (*n* = 3)	Respiratory tract (*n* = 28)	Urine (*n* = 20)	Genitourinary tract (swabs) (*n* = 24)
*C. glabrata*	1	6	4	6
*C. krusei*	2	6	5	5
*C. parapsilosis*	–	4	–	4
*C. albicans*	–	7	7	7
*C. tropicalis*	–	5	4	2

In this study, pure yeasts cultures growing on plate agar were used. They were isolated from clinical samples. This fact confirms that the described method is universal for the correct identification of different strains of *Candida* and allows it to be used as a diagnostic method in clinical laboratories.

Due to the specificity of primers in the PCR, simple detection method, and analyzing of the digestion products profile, the t-RFLP method can probably be used for the identification of *Candida* sp. from biological samples. However, further validation is needed, because the sensitivity of PCR may decrease as a result of the inhibitors, e.g., in blood or sputum (Al-Soud and Rådström 2001; Amicosante et al. 1995).

The sensitivity of the t-RFLP method is also dependent on the restriction products detection method. Capillary electrophoresis is more sensitive than polyacrylamide gel electrophoresis and a smaller amount of digested PCR products is needed. It is also more precise and may be a good solution for routine laboratory diagnostics because it can be automated (Fawley et al. 2015).

## Conclusions

In this study, a novel application for the terminal restriction fragment length polymorphism (t-RFLP) method was developed. The results of this research lead to two main innovative issues: new molecular target for the identification of *Candida* species and showing the potential of the t-RFLP method for diagnostic purposes. This method was successfully applied to bacterial and yeast community investigations in different environments or microbiota analysis. In this study, the genotyping potential of the t-RFLP method has been extended to the identification of *Candida* species. The results of this study yield a novel tool for clinical diagnostics and highlights a new path for the development of the t-RFLP method. The designed method can be a cost-efficient, high-throughput molecular technique able to determine specific and simple restriction patterns for the identification of six clinically important *Candida* species.
